# BeEAM Conditioning including High-Dose Bendamustine before Autologous Stem Cell Transplantation Is Safe and Effective in Patients with Waldenstrom’s Macroglobulinemia

**DOI:** 10.3390/jcm12062378

**Published:** 2023-03-19

**Authors:** Alexander D. Heini, Philipp Beck, Ulrike Bacher, Katja Seipel, Thilo Zander, Michael Daskalakis, Thomas Pabst

**Affiliations:** 1 Department of Medical Oncology, Inselspital, Bern University Hospital, 3010 Bern, Switzerland; 2 Department of Hematology and Central Hematology Laboratory, Inselspital, Bern University Hospital, 3010 Bern, Switzerland; 3 Department for Biomedical Research, University of Bern, 3010 Bern, Switzerland; 4 Division of Medical Oncology, Luzerner Kantonsspital, 6004 Lucerne, Switzerland

**Keywords:** Waldenstrom, macroglobulinemia, BeEAM, high-dose chemotherapy, autologous stem cell transplantation

## Abstract

High-dose chemotherapy (HDCT) with autologous stem cell transplantation (ASCT) is an option to consolidate remission in Waldenstrom’s macroglobulinemia (WM), particularly in selected younger patients with chemosensitive disease. BEAM, consisting of BCNU, etoposide, cytarabine, and melphalan, is often used as a conditioning regimen. However, problems with BCNU, including pneumotoxicity, tolerance, and availability, necessitate the search for alternatives. In this pilot study, we investigated high-dose chemotherapy with BeEAM, in which BCNU is replaced with high-dose bendamustine as an alternative conditioning regimen in six subsequent patients with WM. Bendamustine treatment was well tolerated without unexpected toxicities. The overall response rate was 6/6 patients (2 very good partial responses (VGPR) and 4 PR). After a median follow-up of 72 months, two (33%) patients relapsed. Median progression-free and overall survivals were not reached, and no severe late-onset toxicities were observed so far. In this pilot study, BeEAM conditioning before ASCT seems feasible, safe, and effective in patients with WM.

## 1. Introduction

Waldenstrom’s macroglobulinemia (WM) is a rare B-cell lymphoma entity, which is defined as a lymphoplasmocytic lymphoma with infiltration of the bone marrow with IgM monoclonal gammopathy [1,2]. The age- and sex-adjusted incidence is 0.57 per 100,000 person-years, with males more commonly affected than females [3]. The incidence appears to have remained remarkably stable for decades [3]. Age at initial diagnosis ranges from a median of 63–75 years, but a minority (less than 10%) of patients is younger than 50 years [4]. Median survival is more than 10 years in patients younger than 70 years, approximately 7 years in patients aged 70–79 years, and approximately 4 years in patients older than 80 years [5]. In a retrospective analysis, the survival of WM patients aged 23–55 years was not significantly different to an age- and sex-matched healthy population. However, in this analysis, 63% of patients were considered as IPSS-WM low risk and only a minority (10%) as high risk [6]. Despite the generally indolent course of the disease and improvement in prognosis, the disease remains a major cause of morbidity and mortality in younger patients with intermediate- and high-risk WM and novel strategies are needed, especially for this patient population [7].

The preferred initial therapeutic approach for younger patients who are in need of therapy is a rituximab-based chemoimmunotherapy. Chemotherapy-free regimens such as rituximab monotherapy appear attractive in younger patients, reflecting concerns about secondary malignancies after exposure to cytotoxic chemotherapy. However, there are no randomized survival data to support the benefit of the omission of chemotherapy in this patient population [6]. Second-line options after chemoimmunotherapy include the repetition of first-line therapy followed by high-dose chemotherapy (HDCT) with autologous stem cell transplantation (ASCT). Moreover, ibrutinib, a Bruton’s tyrosine kinase inhibitor (BTKI), has shown impressive activity alone [8] or in combination with rituximab [9] in the second-line setting. Most recently, acalabrutinib [10] and zanubrutinib [11] have emerged as potent alternatives with a favorable safety profile compared with ibrutinib. In younger patients fit for HDCT/ASCT, the choice of optimal therapy or the sequence is unclear, as randomized data are lacking.

HDCT with ASCT is widely used as consolidation treatment in good-risk AML and various types of NHL, and it has been demonstrated to prolong disease-free survival (DFS) and overall survival (OS) in these diseases [12,13,14,15,16,17,18,19,20,21]. In contrast to nonmyeloablative therapies, ASCT can result in increased treatment-associated mortality and morbidity and is therefore applied in young fit patients with few comorbidities [22]. Treatment-associated mortality has been reduced to far less than 10% owning to improved conditioning regimens and supportive care [23].

In WM, HDCT/ASCT is an option for consolidation treatment in younger patients with chemosensitive disease but is also used in patients with early relapse and clinically aggressive disease [4]. In the 1990s and early 2000s, melphalan [24] or thiotepa, busulfan, and cyclophosphamide [25] with or without total body irradiation (TBI) were used as HDCT conditioning regimens. In these reports, mostly partial responses were observed, and treatment-related mortality was high, with one death occurring in four treated patients due to arterial thromboembolism [25]. In a more recent study comprising 158 patients with WM undergoing HDCT/ASCT in first or later remission, Kyriako et al. reported a 5-year OS of 68.5% and PFS of 39.7% [26]. Patients were treated with a variety of chemotherapy- and TBI-based conditioning regimens and nonrelapse mortality was 3.8% after 1 year. Finally, Arulogun et al. reported a median PFS of 4.5 years and estimated 2-year and 5-year PFS rates of 75% and 35.9%, respectively [27]. Patients were treated with lomustine, etoposide, cytarabine and melphalan (LEAM), BEAM, or single-agent melphalan, and the treatment-related mortality during inpatient treatment for HDCT/ASCT was estimated at 6.2%.

In recent years, TBI-based regimens have become less common, and chemotherapy-only HDCT regimens have become the standard of care. Evidence for the superiority of one regimen versus the other is lacking so far [12]. A commonly used conditioning regimen is BEAM, consisting of BCNU (carmustine), etoposide, cytarabine, and melphalan [12,28]. Concerns have risen over the use of BCNU, as it can lead to pulmonary toxicity with pneumonitis and diffuse alveolar damage. Incidence is estimated at <10% when used as a single agent; however, it can be considerably higher when used in combination [12,29,30,31]. Moreover, recently, availability issues have become problematic, together with the poor tolerance of BCNU.

Consequently, there is a need for an effective alternative replacing BCNU. In our study, we evaluated the feasibility, safety, and efficacy of the BeEAM regimen, in which BCNU is replaced by bendamustine. Previous studies have established evidence for the efficacy, safety, and improved toxicity profile of the BeEAM regimen as HDCT for patients with Hodgkin’s lymphoma and NHL [23,32,33]. So far, no data on the use of BeEAM conditioning in WM have been reported. In this retrospective single-center study, we report tolerance and outcome in six consecutive patients with WM treated with BeEAM conditioning followed by ASCT between 2015 and 2022.

## 2. Patients and Methods

### 2.1. Patient Population

In this single-center retrospective study, we analyzed all consecutive patients with WM who were treated with BeEAM conditioning followed by ASCT between 2015 and 2022 at the University Hospital in Bern, Switzerland. All patients gave written informed consent, and this study was approved by the local ethics committee of Bern, Switzerland.

### 2.2. Treatment

200 mg/m^2^ bendamustine was administered on days −7 and −6 as a single 2 h infusion in 500 mL normal saline supported by forced hydration. On days −5 to −2, 200 mg/m^2^ cytarabine and 150 mg/m^2^ etoposide were administered every 12 h as a 30 min infusion each in 500 mL normal saline. Finally, 140 mg/m^2^ melphalan was given as a single one-hour infusion in 500 mL normal saline supported by forced hydration on day −1. At least 2.0 × 10^6^ CD34+ cells/kg body weight (b.w.) were reinfused on day 0. All patients received weight-adapted G-CSF (filgrastim at 5 μg/kg b.w.) starting at day +6 after ASCT until neutrophils exceeded 0.5 × 10^9^/L for three consecutive days. Patients routinely had antiviral (oral acyclovir 500 mg twice daily) and antifungal prophylaxis (oral fluconazole 400 mg once weekly and oral sulfamethoxazole/trimethoprim 800/160 mg three times per week). No antibiotic prophylaxis was given. Hyperuricemia prophylaxis was given from days −7 to −1 with 300 mg daily oral allopurinol. Patients received platelet or red cell transfusions according to local standards (platelet transfusions if platelets < 20 × 10^9^/L and signs of bleeding, <10 × 10^9^/L and fever or <5 × 10^9^/L, and red blood cell transfusions if hemoglobin < 70 g/L). Patients remained hospitalized for the entire procedure, starting with the administration of HDCT, and were dismissed after hematologic and adequate physical recovery.

### 2.3. Measurements and Definitions

Risk classification was based on data before or during first-line therapy using the International Prognostic Scoring System for Waldenstrom’s Macroglobulinemia (ISSWM) [34]. MYD88 mutation status was determined in peripheral blood or bone marrow samples by ddPCR, as previously described [35]. Toxicities were graded according to Common Terminology Criteria for Adverse Events (CTCAE) version 5.0 [36]. The last line of therapy and/or maintenance therapy before HDCT was considered induction therapy. Endpoints of this study were time to progression or relapse of the underlying disease, the occurrence of treatment-associated toxicity, and death from any cause. Overall survival (OS) was defined as the time from ASCT until death from any cause or last follow-up. Progression-free survival (PFS) was defined as the time from ASCT until first relapse/progression, death, or last follow-up, whichever occurred first. Remission status was determined according to the sixth International WM Workshop recommendations [37]. Follow-up after discharge from hospital was performed on day 100 after ASCT and at regular intervals thereafter (every three months in the first year, then every six months, and finally annually) or as clinically required. One hundred days after ASCT, a bone marrow examination was obtained for staging, and in case of lymphadenopathy or organomegaly before treatment initiation, a CT scan was performed. Data on remission status and toxicities were collected until May 2022 (data cut-off of this study).

### 2.4. Statistical Analysis

Survival curves were calculated using the Kaplan–Meier method. *p*-values of <0.05 were considered statistically significant. Analyses were performed using GraphPad Prism Version 9 (GraphPad Software, Inc., La Jolla, CA, USA).

## 3. Results

### 3.1. Patient Characteristics

Details of patient characteristics are summarized in Table 1. All patients happened to be male with a median age of 58 years at ASCT. Four patients were classified as ISSWM low risk, and two were classified as intermediate risk. MYD88 mutations were detected in five out of six patients (83%), and the median bone marrow infiltration at diagnosis was 22.5% (range 5–60%).

The median time to initiation of therapy after initial diagnosis was 3 months, and the median time from diagnosis to HDCT/ASCT was 25.5 months. Patients had a median of two lines of therapy before HDCT/ASCT, with four patients already having been pretreated with bendamustine in combination with rituximab before HDCT. One patient underwent multiple plasma exchanges due to severe hyperviscosity syndrome with macular edema and 90% visual acuity loss. No patient was previously treated with radiotherapy.

First-line therapy consisted of rituximab and bendamustine (RB) in three patients (50%) and rituximab, bortezomib, and dexamethasone (BDR), rituximab, fludarabine, and cyclophosphamide (R-FC), and dexamethasone, cyclophosphamide, and rituximab (DRC) in one patient each. WM was more likely to be refractory in patients with intermediate ISSWM and was consequently treated with more lines of therapy before HDCT compared with the other patients. No patient underwent therapy with a BTKI prior to HDCT. In all patients, a Karnofsky performance score of >70% was documented at the start of HDCT. Two patients had relevant comorbidities, being depression requiring psychiatric treatment in one patient and chronic kidney disease KDIGO G3a in one patient.

### 3.2. Mobilization Treatment, HDCT, and ASCT

Five patients underwent mobilization chemotherapy with vinorelbine/G-CSF [38] and one with gemcitabine/G-CSF [39]. After G-CSF administration, autologous stem cells were obtained from peripheral blood in all patients. CD34+ selection was performed in four patients, and finally, a median of 3.9 × 10^6^/kg b.w. was transfused. All patients underwent HDCT without dose restriction, as described above.

### 3.3. Hematologic Toxicity and Recovery

**Figure 1:**
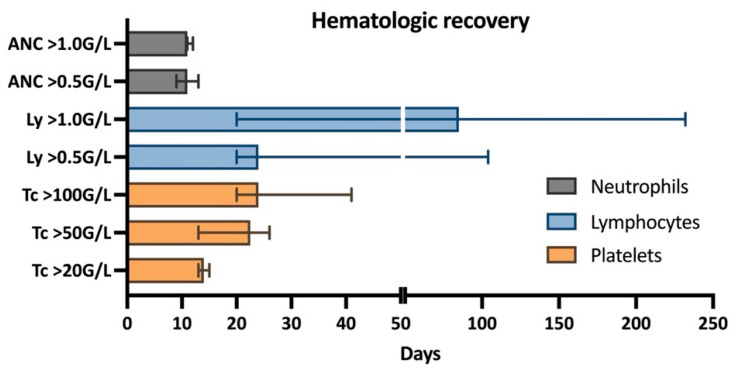
Hematologic recovery: ANC, absolute neutrophil count; Ly, lymphocytes; Tc, platelets.

Details of hematologic toxicity and recovery are reported in Table 2 and Figure 1. The median time to neutrophil recovery >0.5 × 10^9^/L was 11 days (range 11–12). Platelet recovery to 20 × 10^9^/L, 50 × 10^9^/L, and 100 × 10^9^/L was documented after a median of 14 (range, 13–15), 22.5 (range, 13–26), and 24 (range, 20–41) days, respectively. The median time until lymphocyte recovery to 0.5 × 10^9^/L and 1.0 × 10^9^/L was 24 (20–104) and 85 (20–232) days, respectively. Patients required a median of two units of red blood cell transfusions (range 0–5), with only one patient not requiring any transfusion. All patients received platelet transfusions during hospitalization, with a median of three units (range 1–5). Clinical manifestations of thrombocytopenia were observed in four patients with petechial bleedings (three patients) or a spontaneously occurring hematoma (two patients). One patient did not achieve complete hematologic recovery with persistent non-transfusion-dependent anemia of around 115 g/L persisting at the last follow-up.

### 3.4. Infections during Hospitalization

Infectious complications during hospitalization are summarized in Table 3 and Supplemental Tables S1 and S2. Five patients developed neutropenic fever with a median duration of 3 days. The corresponding blood cultures were positive for coagulase-negative *Staphylococci* in three patients, *Klebsiella pneumoniae* in two patients, and *Escherichia coli* and *Bacillus cereus* in one patient each. Two patients showed evidence of multiple pathogens. Patient B additionally tested positive for rhinovirus by nasopharyngeal smear. No fungal infections were documented.

The focus of infection was neutropenic colitis in four patients. Patient E was diagnosed with a central line-associated bloodstream infection (CLABSI). Patient C had marantic parotitis during hospitalization. Patient B met 4/4 SIRS criteria in the setting of a symptomatic sepsis, but responded well to antibiotics, and no admission to the ICU was necessary. All six patients received antibiotic treatment.

### 3.5. Nonhematologic, Noninfectious Toxicities during Hospitalization

We listed the details of nonhematologic, noninfectious toxicities during and after hospitalization in Supplemental Tables S1 and S2. The most frequent toxicities involved the gastrointestinal tract, metabolism, and nutrition. Toxicities reported were loss of appetite, nausea, vomiting, mucositis, and diarrhea. The median weight loss during hospitalization was 3.3 kg (range 0.1–6.2 kg), representing a median loss of 4.3% against baseline weight (range 0.1–7.2%). These toxicities also account for the majority of grade 3/4 toxicities, with severe loss of appetite with subsequent total parenteral nutrition (TPN) being necessary for five patients. Due to TPN, patient E developed hyperglycemia requiring transient insulin support. Patient A developed hypotonic hyponatremia (123 mmol/L), which had to be treated with hypertonic saline and fluid restriction.

Two patients developed acute kidney injury (AKI) during HDCT/ASCT, one patient with previously normal kidney function developed AKI grade 2, and a second patient with a history of chronic kidney disease (KDIGO G3a) due to focal segmental proliferative glomerulonephritis developed AKI grade 1, both corresponding to CTCAE grade 3 toxicity. Both patient’s kidney functions recovered to baseline levels until discharge after ASCT treatment.

### 3.6. Complications after Hospitalization

Patients were discharged after a median duration of 23 days (range 22–25) after ASCT. Within 100 days after ASCT, two patients developed infections, with patient D requiring hospitalization due to clostridium difficile colitis and patient E developing a febrile episode, most likely viral, with cough and CRP elevation but without pathogen identification. Five patients developed infections after 100 days: One developed herpes zoster 8 months after ASCT. Three patients had recurrent upper respiratory infections within the first 2 years after ASCT, with Patient C requiring hospitalization due to pneumonia 6 months after ASCT. The same patient required surgical intervention due to a paramandibular abscess 33 months after ASCT, which occurred after dental root canal treatment. Patient B developed recurrent ENT infections during maintenance therapy with rituximab and was later diagnosed with bilateral bacterial conjunctivitis and prostatitis.

Late-onset toxicities of BeEAM-HDCT were observed in three patients. Patient B reported persistent paresthesia 4 months after ASCT. In addition, secondary IgA and IgG deficiency requiring immunoglobulin substitution was detected 9 months after ASCT. Most recently, an IgG level of 2.7 g/L was measured 57 months after ASCT, requiring ongoing intravenous immunoglobulin substitution. Patient E was found to have a persistent normocytic anemia with fatigue and elevated GGT at 13 months, which was not further investigated. A secondary malignancy was detected in patient F, who developed lentigo maligna melanoma 2 years after HDCT.

### 3.7. Outcome

**Figure 2:**
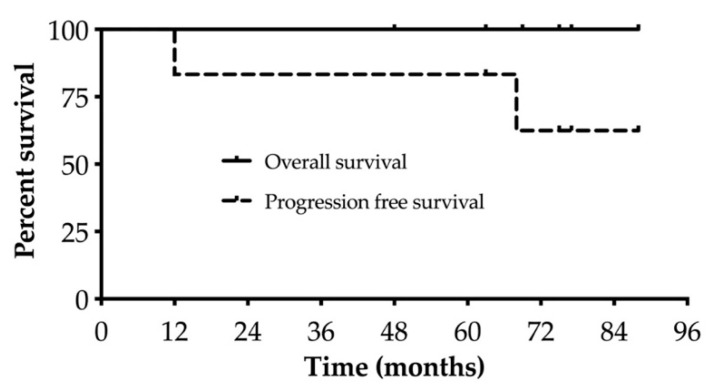
Survival curves.

Outcome data are summarized in Table 1 and Table 4 and Figure 2. One hundred days after ASCT, four patients achieved a partial response, and two patients had a very good partial response (VGPR). Patient A underwent tandem ASCT with melphalan 4 months after HDCT with BeEAM. One patient who achieved PR proceeded to maintenance therapy with rituximab after HDCT/ASCT, which was discontinued due to the onset of hypogammaglobulinemia with recurrent ENT infections. Patient F suffered a subsequent (fourth) relapse after 12 months, which manifested as autoimmune hemolytic anemia and Guillain–Barré syndrome, whereupon the patient received ibrutinib. After the subsequent (fifth) relapse, he was again treated with ibrutinib and was in ongoing PR at the last follow-up. A second patient who underwent HDCT/ASCT as consolidation treatment in the first remission relapsed from PR 68 months after ASCT with an asymptomatic rise in paraprotein levels. As there was so far no indication for therapy, the patient is currently being followed closely. After a median follow-up of 72 months, median overall and progression-free survivals were not reached.

## 4. Discussion

Over the recent decades, the prognosis of patients with WM has steadily improved. However, due to the rarity of WM, the optimal treatment strategy, especially in younger patients, is poorly defined, and the evidence for eventual HDCT/ASCT is primarily based on small retrospective studies. The 2018 ESMO guideline recommendations regarding ASCT refer to the Kyriakou et al. 2010 study with 158 WM patients, in which different HDCT regimens were investigated [4,26]. The present study is the first one to investigate HDCT with BeEAM followed by ASCT in WM patients and provides evidence for its efficacy and tolerability.

One hundred days after BeEAM-HDCT with ASCT, all six patients showed a response ranging from VGPR to PR. One patient who relapsed 12 months after ASCT had intermediate-risk disease and was intensively pretreated, with both conditions indicating an adverse prognostic profile [26]. A second patient showed a late relapse almost 6 years after ASCT. The other four patients remain in partial and very good partial responses at the last follow-up. No treatment-related death occurred. Thus, the response to BeEAM seemed to be favorable in our pilot series of six patients, with achievement of stable remissions in two-thirds of the patients and with median OS and PFS not being reached after a follow-up of 6 years. In their 2010 paper, Kyriakou et al. demonstrated 5-year PFS and OS rates of 39.7% and 68.5% [26]. In our analysis, this number is higher, owning to improved options for later-line therapy. The authors noted a nonrelapse mortality of 3.8% after 1 year and 5.6% after 5 years, which is lower than what was noted in the Arulogun study, in which inpatient nonrelapse mortality was estimated at 6.2% [27]. A total of 6.3% of patients in the Kyriakou cohort developed (mainly hematologic) secondary malignancies, indicating considerable toxicity of the regimens in use in the 1990s and early 2000s [26].

In our cohort, acute hematologic toxicity was within the expected range, and recovery was similar to previous reports on WM patients treated with BEAM [40] and patients with various lymphomas treated with BeEAM [23]. Only the interval to lymphocyte recovery was slightly longer, with a median of 24 days, probably due to the lymphodepleting potential of bendamustine [41], and similar to what was documented after BeEAM-HDCT in patients with various lymphomas [23].

As expected, all patients developed neutropenic fever. The pathogen spectrum of the documented infections was comparable to what is described in the literature in HDCT patients with neutropenia [42]. The only infection requiring hospitalization after day 100 was a paramandibular abscess after dental surgery in one patient. A prolonged or increased susceptibility to infections after BeEAM-HDCT, especially triggered by immunoglobulin deficiency, needs to be investigated in further studies. However, immunoglobulin substitution seems to be warranted, as a reduction in the severity and incidence of recurrent upper respiratory tract infections/ENT infections was observed in the two patients treated with intravenous immunoglobulin (IVIG) for secondary immunoglobulin deficiency.

Nonhematological toxicities during hospitalization were mostly within the expected acute onset toxicity of myeloablative therapy/ASCT and were completely reversible during the therapy. It should be emphasized that two of the six patients developed acute renal failure (one AKI grade 1, one AKI grade 2). Both were fully reversible during the inpatient stay and required no hemodialysis. This corresponded to our previous observations in a cohort of 122 patients with NHL and myeloma undergoing bendamustine-based high-dose chemotherapy [23,43]. Moreover, 51 of 122 patients showed acute kidney injury of any grade, and transient hemodialysis was necessary for three patients. As also reported previously, nephrotoxicity was reversible in the vast majority of patients [43]. In our cohort, no long-term renal toxicity was found. No other grade 3 or 4 acute onset toxicities were found in the present case series, suggesting no unexpected toxicity of BeEAM-HDCT in WM patients.

Late-onset toxicity was within the expected range after myeloablative therapy. One patient developed IgA/IgG hypogammaglobulinemia, which may be explained as a side effect of BeEAM-HDCT but could also be a consequence of maintenance therapy with rituximab [44,45]. No pulmonary or cardiac late-onset toxicity was documented. One patient developed lentigo maligna melanoma around 2 years after BeEAM-HDCT. As secondary skin cancers after HDCT almost exclusively present as nonmelanoma skin cancers, a relationship with BeEAM therapy seems unlikely [46,47].

Obvious limitations of this study are the sample size and its retrospective character, and post-transplant monitoring was performed in the patients in part in cooperating hemato-oncologic centers. All toxicities were examined and classified retrospectively. ISSWM grading and remission status also had to be determined retrospectively for most patients, whereby comprehensive serum protein electrophoresis results throughout the study period were missing in some cases. Due to the limitations mentioned above and the lack of a control group, no conclusive statements can be made regarding the efficacy and long-term effects of BeEAM-HDCT. However, our data indicate a favorable outcome and manageable safety profile in WM patients, and given the rarity of WM and the rather rare indication for HDCT/ASCT for this indication, the cohort seems relevant, although being limited by number.

The approval of ibrutinib and other BTKI has opened new possibilities for the treatment of patients with WM. They have been shown to achieve high response rates and durable remissions in both treatment-naïve and pretreated patients, especially in the CXCR4 wild-type subgroup [48]. Side effects of BTKI are mainly infections and generally mild in grade and manageable with dose reductions or interruptions. However, grade 3 and 4 bleeding and atrial fibrillation are known to occur in around 1–5% of patients [6,8,9,10,11]. The optimal choice of therapy and sequence of therapy lines is not known, especially in younger fit patients that qualify for HDCT/ASCT. When treating chronic disease in a younger patient, the choice of a continuous tablet-based therapy may appear unappealing. These patients may benefit from HDCT/ASCT, as it offers the chance for long treatment-free intervals. Our data support the use of HDCT/ASCT in this patient cohort with deep and durable responses and a manageable safety profile. Careful selection of patients for this intensive treatment is crucial to improve outcomes and minimize the risk of toxicity. Still, head-to-head comparisons between BTKI and HDCT/ASCT are lacking and will need to be explored in further larger multicenter prospective studies.

## 5. Conclusions

In this pilot patient series, we demonstrate that younger patients with low- and intermediate-risk WM can benefit from BeEAM-HDCT and ASCT with long-lasting responses. No new or unexpected safety signals emerged. Open questions remain, especially regarding the optimal sequence of therapies and patient selection. These questions should ideally be addressed in large multicenter studies.

## Figures and Tables

**Table 1:** Summary of patient’s baseline characteristics, therapy before HDCT/ASCT, and response to HDCT/ASCT.

	Patient A	Patient B	Patient C	Patient D	Patient E	Patient F	Median
**Baseline characteristics**							
Age at diagnosis (years)	45	42	52	58	61	57	54.5
ISSWM risk group	Intermediate	nd	Good	nd	Good	Intermediate	
MYD88 status	mutated	wild type	mutated	mutated	mutated	mutated	
**Therapy before HDCT/ASCT**							
Months from initial diagnosis to first therapy	0	15	3	65	3	1	3
First line	R-FC	R-Benda	DRC	BDR	R-Benda	R-Benda	
Second line	R-CHOP	R-VCD	R-Benda			Ofatumumab	
Third line	BDR	-	-	-	-	Benda	
Fourth line	-	-	-	-	-	Ofatumumab	
Lines before HDCT	3	2	2	1	1	4	2
**At HDCT/ASCT**							
Remission status at HDCT/ASCT, response to last line of therapy	SD	MR	PR	MR	PR	SD	
Months from initial diagnosis to HDCT	8	21	30	68	10	76	25.5
Age at HDCT (years)	45	44	54	64	62	64	58
**After HDCT/ASCT**							
Best response to HDCT/ASCT	VGPR	PR	VGPR	PR	VGPR	VGPR	
Post-ASCT therapy	Tandem ASCT	Rituximab Maintenance	-	-	-	-	
Progression	No		No	Yes, +68 mt	No	Yes, +12 mt	
Postprogression therapy	-	-	-	None	-	Ibrutinib	

Abbreviations: nd, no data; HDCT, high-dose chemotherapy; ASCT, autologous stem cell transplantation; R-FC, rituximab/fludarabine/cyclophosphamide; R-Benda, rituximab/bendamustine; DRC, dexamethasone/rituximab/cyclophosphamide; BDR, bortezomib/dexamethasone/rituximab, R-CHOP, rituximab/cyclophosphamide/doxorubicin/vincristine/prednisolone; R-VCD, rituximab/bortezomib/cyclophosphamide/dexamethasone; SD, stable disease; MR, minimal response; PR partial response; VGPR, very good partial response.

**Table 2:** High-dose chemotherapy and hematologic toxicity.

**BeEAM chemotherapy given, n (%)**	6 (100%)
Full dose given as planned, n (%)	6 (100%)
**Transplanted CD34+ cells**, median, × 10^6^/kg, (range)	3.9 (2.4–4.5)
CD34+ selection, n (%)	4 (67%)
**Median time to engraftment**, days (range)	
Platelets > 20 × 10^9^/L	14 (13–15)
Platelets > 50 × 10^9^/L	22.5 (13–26)
Platelets > 100 × 10^9^/L	24 (20–41)
Lymphocytes > 0.5 × 10^9^/L	24 (20–104)
Lymphocytes > 1.0 × 10^9^/L	85 (20–232)
Neutrophils > 0.5 × 10^9^/L	11 (9–13)
Neutrophils > 1 × 10^9^/L	11 (11–12)
**Transfusions**, units, median (range)	
Red blood cells	2 (0–4)
Platelets	3 (1–5)
**Hospitalization**, days, median (range)	23 (22–25)

**Table 3:** Infectious complications.

At least one febrile episode of ≥38.0 °C, n (%)	5 (83%)
Median days with fever, range	3 (2–8)
Identified pathogens, n (%)	4 (67%)
Patients with positive blood cultures, n (%)	4 (67%)
Gram-positive bacteria, n (%)	4 (67%)
Gram-negative bacteria, n (%)	2 (33%)
Viral infection, n (%)	1 (17%)
Fungal infection, n (%)	0 (0%)
Multiple pathogens identified, n (%)	2 (33%)
Antibiotic treatment of infection, n (%)	6 (100%)

**Table 4:** Outcome.

Follow-up, median, months (range)	72 (48–87.5)		
OS, median, months (range)	n.r.		
PFS, median, months (range)	n.r.		
Relapse, n (%)	2 (32%)		
Deaths, n (%)	0		
**Remission status**	**Before ASCT (day 0)**	**At day 100**	**At last follow-up**
CR	0	0	0
VGPR	0	2 (34%)	3 (50%)
PR	2 (33%)	4 (66%)	1 (17%)
MR	2 (33%)	0	0
SD	2 (33%)	0	0
PD	0	0	2 (33%)

Abbreviations: n.r., not reached; OS, overall survival; PFS, progression-free survival; CR, complete response; VGPR, very good partial response; PR, partial response; MR, minor response; SD, stable disease; PD, progressive disease.

## Data Availability

Data supporting the findings of this manuscript are available from the corresponding author upon request.

## Supplementary Materials

The following supporting information can be downloaded at: https://www.mdpi.com/article/10.3390/jcm12062378/s1, Table S1: Toxicities according to CTCAE 5.0, Table S2: Toxicities according to patient.
