# Supplementary material for: BeEAM Conditioning including High-Dose Bendamustine before Autologous Stem Cell Transplantation Is Safe and Effective in Patients with Waldenstrom’s Macroglobulinemia
Source: J Clin Med. 2023 Mar 19;12(6):2378. doi: 10.3390/jcm12062378 (PMC10057504; doi:10.3390/jcm12062378)
Supplement: Supplementary file 1 [file jcm-12-02378-s001.zip › jcm-2246927-supplementary.pdf]

Supplementary Table S1. Toxicities according to CTCAE 5.0.

Type of toxicity according to CTCAE 5.0	Number of patients with adverse event, n (%)	Grade	
		1 – 2	3 – 4
**Ear and labyrinth disorders**	1 (17%)	Hearing impaired (1)	
Endocrine disorder	1 (17%)	SIADH (Other, specify) (1)	
Eye disorders	1 (17%)	Photophobia (1)	
Gastrointestinal disorders	6 (100%)	Dry mouth (2), nausea (3), vomiting (3), oral/rectal mucositis (3), oral pain (2), diarrhea (5), abdominal pain (2), hemorrhoids (1), hemorrhoidal bleeding (1)	
General disorders and administration site conditions	5 (83%)	Edema (4), fatigue (4)	
Investigations	3 (50%)	Blood antidiuretic hormone abnormal (1), creatinine increased (2)	
Metabolism and nutrition disorders	6 (100%)		Hyperglycemia (1), Anorexia (5), Hyponatremia (1)
Musculoskeletal and connective tissue disorders	1 (17%)	Back pain (1)	
Nervous system disorders	1 (17%)	Paresthesia (1)	
Renal and urinary disorders	2 (33%)		Acute kidney injury (2)
Respiratory, thoracic and mediastinal disorders	1 (17%)	Dyspnea (1)	
Skin and subcutaneous tissue disorders	4 (66%)	Alopecia (4), rash maculo-papular (1)	
**Late onset toxicities**			
Blood and lymphatic system disorders	3 (50%)	Anemia (1), IgA/IgG deficiency (Other, specify) (1)	
Investigations	1 (17%)	GGT increased (1)	
Nervous system disorders	1 (17%)	Paresthesia (1)	

Supplemental Table S2. Toxicities according to patient.

	Patient A	Patient B	Patient C	Patient D	Patient E	Patient F
**Complications during inpatient treatment**						
Neutropenic fever	Yes	Yes	No	Yes	Yes	Yes
Neutropenic colitis	No	Yes	Yes	Yes	No	Yes
Febrile episodes	1	2	0	1	2	2
Days with fever	2	8	0	2	6	4
Spontaneous bleeding	Petechiae	No	Petechiae	Petechiae	Petechiae	No
**Infectious Complications after discharge**						
Infections	No	Otitis media, pansinusitis +9mt	Recurring URTI, Pneumonia +6mt	C. difficile colitis, +2mt	URTI, +10mt	Fever, no focus, +2mt Herpes Zoster, +8mt
Documented Ig deficiency	No	Yes	Yes	No	No	No
Ig Substitution	No	Yes	Yes	No	No	No
**Late onset toxicities**						
Secondary malignancies	No	No	No	No	No	Lentigo Maligna melanoma, +24mt
Other	No	Paraesthesia	No	No	GGT elevation	No

Abbreviations: URTI, upper respiratory tract infection; mt, months; GGT, gamma guanyltransferase
